# Stage-specific nutritional management and developmental programming to optimize meat production

**DOI:** 10.1186/s40104-022-00805-0

**Published:** 2023-01-03

**Authors:** Liang Zhao, Xiangdong Liu, Noe A Gomez, Yao Gao, Jun Seok Son, Song Ah Chae, Mei-Jun Zhu, Min Du

**Affiliations:** 1 College of Animal Science and Technology, Nanjing Agricultural University, 210095 Nanjing, PR China; 2 Nutrigenomics and Growth Biology Laboratory, Department of Animal Sciences, Washington State University, WA 99164 Pullman, USA; 3 Laboratory of Perinatal Kinesioepigenetics, Department of Obstetrics, Gynecology and Reproductive Sciences, University of Maryland School of Medicine, MD 21201 Baltimore, USA; 4 School of Food Science, Washington State University, WA Pullman, USA

**Keywords:** Adipose tissue, Embryonic development, Fetal programming, Fibro/adipogenic progenitors, Marbling, Nutritional regulations, Skeletal muscle

## Abstract

Over the past few decades, genetic selection and refined nutritional management have extensively been used to increase the growth rate and lean meat production of livestock. However, the rapid growth rates of modern breeds are often accompanied by a reduction in intramuscular fat deposition and increased occurrences of muscle abnormalities, impairing meat quality and processing functionality. Early stages of animal development set the long-term growth trajectory of offspring. However, due to the seasonal reproductive cycles of ruminant livestock, gestational nutrient deficiencies caused by seasonal variations, frequent droughts, and unfavorable geological locations negatively affect fetal development and their subsequent production efficiency and meat quality. Therefore, enrolling livestock in nutritional intervention strategies during gestation is effective for improving the body composition and meat quality of the offspring at harvest. These crucial early developmental stages include embryonic, fetal, and postnatal stages, which have stage-specific effects on subsequent offspring development, body composition, and meat quality. This review summarizes contemporary research in the embryonic, fetal, and neonatal development, and the impacts of maternal nutrition on the early development and programming effects on the long-term growth performance of livestock. Understanding the developmental and metabolic characteristics of skeletal muscle, adipose, and fibrotic tissues will facilitate the development of stage-specific nutritional management strategies to optimize production efficiency and meat quality.

## Introduction

Over time, contemporary livestock breeds have been extensively selected for rapid lean growth and feed efficiency, which resulted in substantial increases in meat production efficiency during the past few decades [1, 2]. Because adipose metabolism is energetically costive, optimizing growth efficiency coincides with reduction in fat deposition [3]. However, meat quality of fast-growing livestock is often compromised, including the reduction in intramuscular fat (“marbling” in beef cattle) and the enlargement of muscle fibers, which collectively decreased tenderness, juiciness, and flavor [4, 5]. Similarly, excessive hypertrophy of muscle fibers disrupts metabolic phenotypes of muscle fibers, including an increase in glycolytic white muscle fibers which influence the processing functionality of meat [6].

Ruminant animals, including sheep and cattle, frequently experience nutrient shortages during their pregnancy caused by seasonal variations, frequent droughts, and other unfavorable geological locations [7]. Compared with the central nervous system and other vital organs, skeletal muscle and adipose tissue have low priority in nutrient partitioning, rendering them susceptible to nutritional fluctuations [7, 8]. Maternal nutrient deficiency impairs the early development of progeny, exerting long-term effects on the composition and metabolism of skeletal muscle and adipose tissue in offspring [7, 9]. Therefore, proper maternal nutritional management improves the development of livestock conceptuses and their subsequent production efficiency.

Fundamental events of mammalian development occur during the embryonic, fetal, and neonatal stages [7, 9, 10]. While differentiation of pluripotent stem cells and organogenesis occur during the embryonic stage, most tissues and organs mature during the fetal stage [11]. Late fetal and neonatal stages are important for the growth of most tissues and the formation of tissue-resident stem cells, such as satellite cells and fibro/adipogenic progenitors [12, 13]. Recent studies in maternal effects on animal development have extensively focused on the fetal stage between the second to third gestation [14–17]. For example, maternal nutrient deficiency during the fetal stage results in impaired fetal development with long-term consequences on offspring performance, referred to as “fetal developmental programming” [18]. In addition, limited but growing studies show that the embryonic stage during the very early gestation is another critical window for developmental programming [19, 20].

In this review, we summarize recent advances in the development of skeletal muscle, adipose tissue, and connective tissues, the effects of maternal nutrition on the early embryonic and fetal development, and their impacts on body composition and meat quality of offspring. Understanding the developmental patterns and intrinsic mechanisms behind tissue development in livestock will facilitate the development of stage-specific maternal nutritional regimens that can optimize production efficiency and meat quality.

## Developmental programming at the embryonic stage

### Embryonic organogenesis

Mammalian organogenesis initiates after the formation of three embryonic germ layers (i.e., ectoderm, endoderm, and mesoderm) during gastrulation [21], which is followed by the formation of major organs and tissues, establishing a blueprint for fetal and postnatal development. Among them, precursors formed within the mesoderm contribute to the formation of body tissues including the heart, bone, skeletal muscle, adipose, and connective tissues [22, 23]. In developing embryos, the mesoderm is gradually specified into four major subtypes along a mediolateral axis including chordamesoderm, paraxial mesoderm, intermediate mesoderm, and lateral plate mesoderm (LPM).

The chordamesoderm is located at the center region of the developing organism and develops into the notochord. This structure induces the patterning of the neural tubes, establishing the anterior-posterior body axis in early embryos [22, 23]. The paraxial, intermediate, and lateral plates of the mesoderm are located on both sides of the notochord, ordered from medial to more lateral regions. Progenitor cells from the intermediate mesoderm give rise to the urogenital system, which includes the kidney and gonads, while progenitors of the LPM form the cardiovascular system, blood cells, smooth muscle lineages, and parts of the appendicular skeleton [24, 25]. Progenitors of the paraxial mesoderm give rise to the majority of the musculoskeletal system on the dorsal surface, ventral body wall, and limb regions [26].

Recent studies have identified derivatives of the paraxial and lateral plate mesoderm as major sites for embryonic myogenesis and depot-specific adipogenesis, though the detailed biological events and underlying regulatory mechanisms remain poorly understood. Here, we summarize the latest knowledge about the developmental origins of adipose tissue and skeletal muscle, and the correlations of these earliest biological activities with body composition and meat production of offspring.

### Embryonic myogenesis

**Fig. 1:**
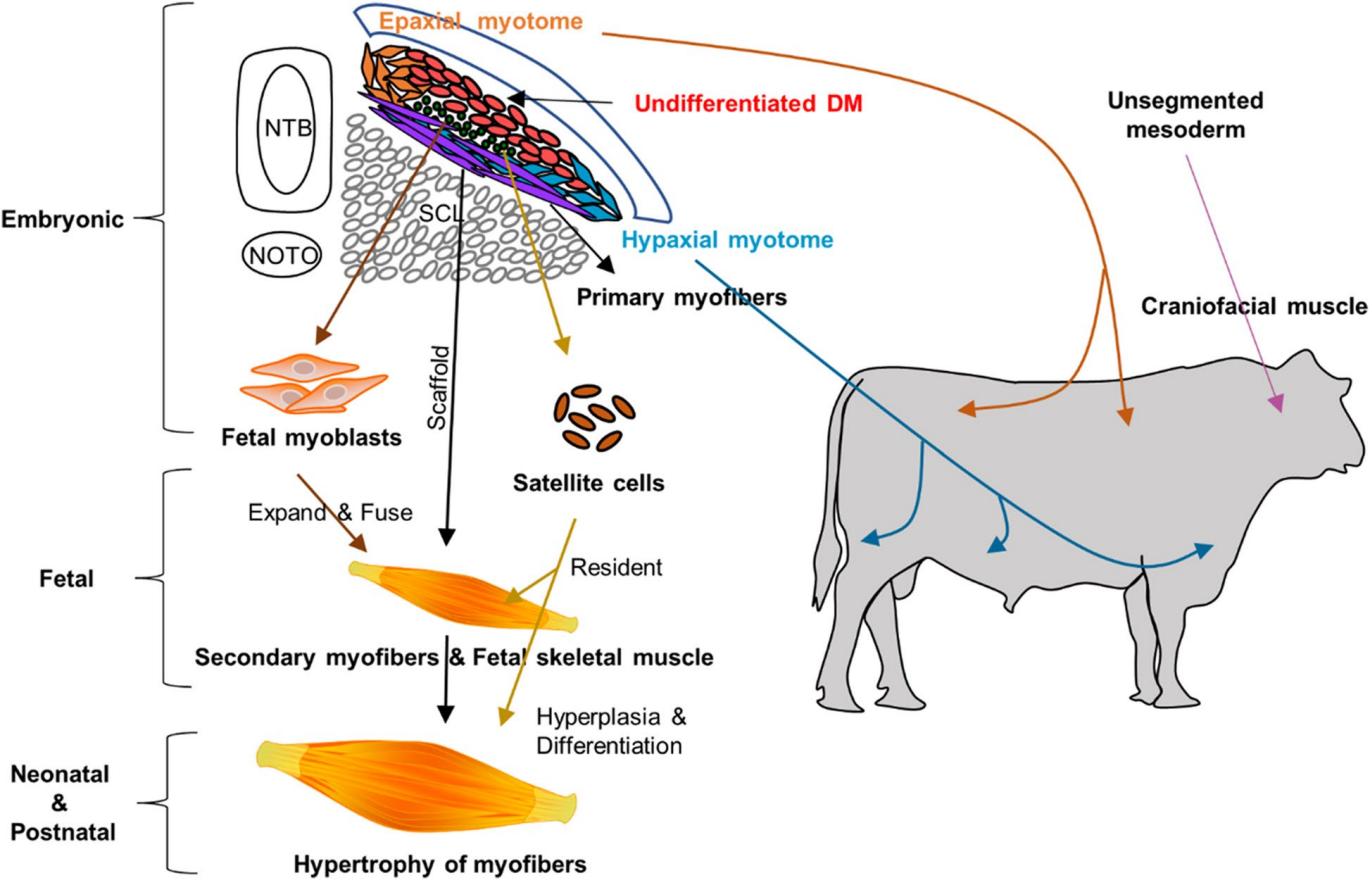
The early development of skeletal muscle in vertebrates (based on rodent models). At the early embryonic stage, mature somites differentiate into the ventral sclerotome (SCL) and the dorsal dermomyotome (DM). Myogenic progenitors derived from the DM give rise to most of the skeletal musculature except craniofacial muscles which are formed from unsegmented mesoderm. Cells from the DM delaminate downward to form a layer of myotome, which is further divided as epaxial or hypaxial myotome depending on their location. Cells from the epaxial myotome give rise to the deep muscle on the dorsal surface, while cells from the hypaxial myotome develop into muscle in the ventral body wall and limbs. Primary myofibers are formed within the myotome and function as scaffolds for myogenesis at fetal and postnatal stages. In addition, Pax7^+^ fetal myoblasts and satellite cells are also formed at this stage which remain undifferentiated until the fetal stage. Later, fetal myoblasts expand rapidly and fuse with primary myofiber to construct fetal myofibers while satellite cells resident within the interstitial spaces of myofibers at the late stage of gestation. Most myofibers are formed at the prenatal stage and there is only growth in the size but not number of myofibers contributed by satellite cells after birth. Note: The arrows indicate the developmental relationships between myogenic progenitors and skeletal muscle at different locations. NTB: Neural tubes; NOTO: notochord

The paraxial mesoderm is progressively elongated along the anterior-posterior body axis of the vertebrate embryos [23]. In most anterior regions, the paraxial mesoderm is periodically segmented into paired blocks of cells, termed “somite(s)”, on each side of the notochord through a process of “somitogenesis”. Thereafter, mature somites differentiate into the ventral mesenchymal sclerotome (SCL) and the dorsal epithelial dermomyotome (DM), which give rise to the majority of the musculoskeletal system on the dorsal surface, ventral body wall, and appendicular regions [17]. While progenitors within the SCL develop into the vertebral and rib cartilage, myogenic progenitors (MPs) derived from the DM give rise to most of the skeletal musculature, except craniofacial muscles which are formed from unsegmented mesoderm at the anterior end of the body axis (Fig. 1) [27].

Embryonic myogenesis, also known as primary myogenesis, is the initial event of myogenesis during animal development [28–30]. *Pax3* is widely expressed in the dermomyotome and Pax3^+^ cells from the dorsomedial lip delaminate downward to form a layer of myotome. The earliest mononucleated myocytes are formed within the myotome, and they elongate to span the entire length of somites [30, 31]. After that, Pax3^+^/Myf5^+^ cells from the lips of the DM also migrate to the myotome and fuse with existing myocytes to build the primary myofibers which express slow myosin heavy chains (MHCs). A myotome is divided into the epaxial and hypaxial myotome by their locations. Cells from the epaxial myotome give rise to the deep muscle on the dorsal surface, while cells from the hypaxial myotome develop into muscle in the ventral body wall and limbs [32].

Fibroblast growth factor (FGF) signaling produced by the myotome induces the maturation and epithelial-to-mesenchymal transition of the central DM [31, 33–35]. After this reorganization, myogenic precursors for fetal and postnatal stages are formed within this mesenchymal pool of DM. A portion of these precursors become fetal myoblasts, which downregulates *Pax3* but upregulates *Pax7*, and remain undifferentiated until the fetal stage [36]. Pax3^+^/Pax7^–^ embryonic myoblasts also switch to fetal myoblasts through the expression of *Nfix* [37]. Another small portion of these Pax7^+^ precursors remain undifferentiated even after birth and become tissue-resident stem cells (satellite cells), and are responsible for postnatal muscle growth [31].

In summary, the myogenic precursors formed during the embryonic stage not only lead to the formation of primary myofibers, but also contribute to myogenesis during the fetal and postnatal stages. Furthermore, although the number of primary myofibers is limited, they serve as a scaffold for myogenesis during the fetal stage. Therefore, embryonic myogenesis plays fundamental roles in the development and growth of skeletal muscle in offspring.

### Embryonic adipogenesis

Adipose tissues are heterogeneous in terms of their anatomical locations and metabolic characteristics [38]. A primary function of white adipose tissue is the storage of energy as triacylglycerols. Meanwhile, brown adipose tissue has the function of catabolizing lipids for heat production. The distribution of white adipose tissue is primarily divided into the subcutaneous and visceral regions, whereas brown adipose tissue is mainly found in the interscapular and thoracic regions of the body. Our current understanding of embryonic adipogenesis remains quite limited, but technological advances in lineage-tracing and fate-mapping studies have identified the embryonic origins of adipose tissues with depot-specific characteristics [38, 39].

**Fig. 2:**
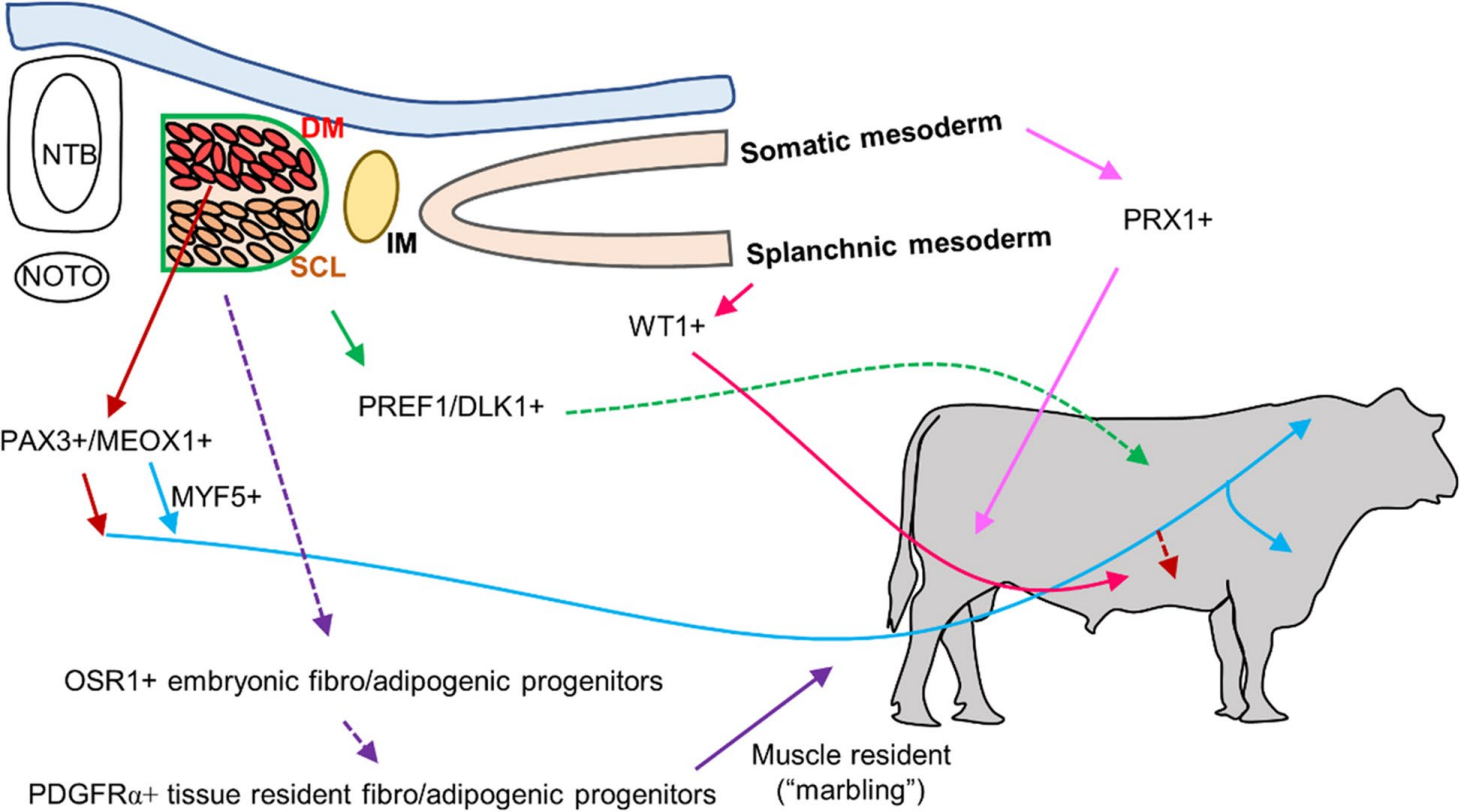
The developmental origins of white adipose tissues in vertebrates (based on rodent models). The development of white adipose tissue shows dot-specific characteristics. Myf5^+^ progenitors from the dermomyotome (DM) of somites give rise to subcutaneous adiposes tissues located in the dorsal-anterior body axis including anterior, interscapular, and retroperitoneal fat. Consistently, Pax3^+^ or Meox1^+^ progenitors within the somites largely overlap with the MYF5 lineage in their contributions to subcutaneous fat, but they may also contribute to the formation of visceral adipose tissues at the perigonadal depot. In addition, Pref1/Dlk1^+^ mesenchymal stem cells from the somites also give rise to the subcutaneous fat. Moreover, PRX1^+^ progenitors in the somatic layer of lateral plate mesoderm (LPM) develop into limb-associated subcutaneous adipocytes especially those at the posterior depots. On the other hand, Wilms tumor 1 (WT1)^+^ embryonic mesothelial cells from the splanchnic layer of LPM develop into the visceral adipocytes. As for tissue-resident fibro/adipogenic progenitors (FAPs) in the skeletal muscle, they have a developmental origin of OSR1^+^ progenitors formed at the mid-gestation. FAPs proliferate at the fetal stage and differentiate into mature intramuscular adipocytes mainly at the fattening stage of beef cattle. Note: NTB: Neural tubes; NOTO: notochord; SCL: sclerotome; IM: intermediate mesoderm

The progenitors formed within the DM differentiate not only into precursors for the skeletal muscle but also for brown, subcutaneous, and visceral white adipose tissues at specific depots (Fig. 2) [40]. Besides skeletal muscle, brown adipose tissue also derives from the same pool of MYF5^+^ progenitors within the DM, and PRDM16 determines the specification of a brown adipogenic lineage [40–42]. Interestingly, these MYF5^+^ progenitors also develop into subcutaneous white adipose tissues at the dorsal-anterior body axis including anterior, interscapular, and retroperitoneal fat [40, 42, 43]. In addition, PAX3^+^ or MEOX1^+^ progenitors within the somites largely overlap with the MYF5 lineage in their contributions to brown and subcutaneous fat, while they additionally develop into over 50% of the visceral white adipose tissues at the perigonadal location in the male, but not female mice [40, 42]. Another study has identified Pref1/Dlk1^+^ mesenchymal stem cells as the earliest precursors for subcutaneous fat, which appear as early as embryonic 10.5 d (E10.5) and mature as lipid-laden adipocytes at E17.5 in mice [44]. Noticeably, *Pref1*/*Dlk1* was also reported to be extensively expressed within the DM and SCL at E10.5 mice [45]. Therefore, the somites, especially of the DM partition, are probably the major sites for embryonic adipogenesis of brown, subcutaneous, and visceral (perigonadal) fat.

In addition to the DM, another derivative of mesoderm, the LPM, also forms precursors for adipose tissue (Fig. 2) [24, 25]. The LPM is gradually divided into two layers, the somatic and splanchnic layers [24, 25]. While cells from the somatic mesoderm differentiate into the skeleton of the pelvic and mesenchymal components of the limb, cells from the splanchnic mesoderm mainly give rise to the circulatory system [24, 25]. Moreover, recent studies found that PRX1^+^ progenitors in the somatic layer develop into limb-associated subcutaneous adipocytes, especially at the posterior depots [46–48]. In addition, some visceral adipocytes were found to descend from Wilms tumor 1 (WT1)^+^ embryonic mesothelial cells [49], which are mostly found in the splanchnic layer [50], though it is also expressed in cells derived from the intermediate mesoderm [51]. Therefore, cells from the LPM contribute to the development of subcutaneous adipose tissue in limb regions, and the majority of visceral adipose tissues.

To summarize, brown adipocytes are generally derived from precursors formed within the DM. Subcutaneous adipose tissue residing in the anterior dorsal region is also derived from the DM, while the tissue residing in the anterior ventral and posterior regions is derived from the somatic layer of the LPM. For visceral fat, a large proportion of perigonadal fat comes from the DM of the male but not female mice, while other comes from WT1^+^ precursors formed within the splanchnic layer of the LPM. Overall, our understanding of embryonic adipose tissue development remains very limited, and more studies are in need to elucidate the regulatory mechanisms for the early specifications of adipogenic lineages.

### Maternal nutrition regulates embryonic muscle and adipose development

During the past few decades, maternal nutrition and developmental programming studies of both animals and humans have focused extensively on the last two-thirds of gestation, when is the critical stage for fetal development [11]. Recently, an increasing number of studies suggest that the period immediately preceding and after conception is also important for developmental programming [52]. This period is referred as the periconceptional period including stages of gamete maturation, fertilization, and development of early embryos. In human and animal models, paternal or maternal malnutrition induces epigenetic and cellular changes in gametes, which alters early embryonic development and predisposes metabolic and neurological diseases in offspring [52–54].

In beef cattle, maternal nutritional studies during the periconceptional period are primarily conducted 60 d pre- and post-breeding [10, 55]. In beef cattle, the first 50 d of gestation (dG) are considered as the embryonic stage [56]. Blastocysts form at 7–8 dG in beef cattle, while embryonic organogenesis starts at about 28 dG and continues until 45 to 50 dG [10]. The fetal stage initiates after 50 dG, when newly formed organs gradually mature into functional organ systems. Sheep share a similar timeline of embryonic development [10, 57]. Studies have found that both underfeeding and overfeeding of ewes before conception results in decreased oocyte quality, which compromises fertilization rate and delayed formation of the blastocysts, in vitro [58–60]. Moreover, nutritional restriction for 6 d immediately post-conception in beef heifer also delayed the formation of blastocysts, suggesting that immediate nutritional changes post-insemination impairs early embryonic development [61]. In addition, high energy-induced maternal obesity before pregnancy increases adipose mass, while impairing the insulin sensitivity of skeletal muscle in 4-month-old offspring lambs [62, 63]. Moreover, a series of studies in beef heifers showed that maternal nutrition during the first 50 dG induces profound changes in the gene expression of transporters for amino acids, glucose, and fructose in uteroplacental tissues, in addition to the changes in amino acid and glucose concentrations in maternal and fetal fluids, which generates long-term effects on fetal and postnatal development [64–67].

Interestingly, moderate maternal nutrient restriction during the first 50 d of pregnancy in heifers induced dramatic changes in gene expressions of the liver and hind limb muscle of the conceptus at 50 dG [19, 20]. Considering the importance of liver in the de novo lipogenesis of ruminant animals, these early changes in metabolic pathways of the liver might alter the subsequent adiposity of offspring. In the skeletal muscle, genes in multiple processes of myogenesis and metabolism of activities were altered [19, 20]. Primary myofibers form between 21 and 60 dG in cattle [68]. Impairments of myogenesis at this stage generate long-term effects on their progeny. Consistently, 60% nutrient deficiency during 30 to 85 dG of beef cattle increased the cross-sectional areas of fetal myofibers, but reduced the density of PAX7^+^ myogenic progenitors [69]. In addition, it decreased IGF1 but increased IGF2 levels, while the expression of genes related to collagen crosslinking was elevated in 85 dG muscle samples [69]. Additionally, 50% nutrient deficiency in ewes 6 d before and 7 d after mating enhanced the size of fetal myofibers, but reduced the ratio of secondary to primary myofibers at 90 dG [70]. Furthermore, maternal over-nutrition induced the accumulation of collagen and expression of *Tgfβ* and *Col3a1* in the fetal skeletal muscle of cattle [71].

Up to now, however, the underlying mechanisms of nutritional effects on embryonic development remain poorly studied. This is partially caused by the very small sizes and immaturity of early embryos. Using advanced techniques of single-cell RNA sequencing, we have identified and characterized myogenic precursors at various differentiating stages within the DM of mice at E9.5, the initial stage for organogenesis [72]. We found that maternal obesity-induced systemic hypoxia enhanced BMP signaling transduction within the DM, which suppressed the expression of *Mef2c*, a critical transcription factor for myofiber differentiation and metabolism. We further propose that the impaired formation of embryonic myofibers and metabolic changes in fetal myoblasts might explain the impaired skeletal muscle development and oxidative metabolism in offspring caused by maternal over-nutrition.

In summary, growing evidence suggests that a critical developmental window during early pregnancy is sensitive to maternal nutritional fluctuations, and is closely associated with tissue development and offspring growth. More studies are needed to elucidate the underlying mechanisms regulating the early myogenic and adipogenic activities of embryos affected by maternal nutrition.

## Developmental programming during fetal and neonatal stages

### Fetal and postnatal myogenesis

Pax3^+^/Pax7^+^ myoblasts formed during embryonic stages remain undifferentiated until the fetal stage [36]. These fetal myoblasts proliferate, differentiate, and fuse to form secondary muscle fibers [73]. The transcription factor *Nfix* is activated by *Pax7* during the fetal stage, which suppresses the expression of embryonic slow MyHC and promotes the expression of fetal specific fast MyHC [37]. Then, myofibers gradually increase their diameter and length by synthesizing new myofibrillar proteins. The majority of myofibers are formed during the fetal stage [74]. Therefore, the number of myogenic precursor cells formed during these stages determines the total number of myofibers in animals, which are critical for muscle growth after birth.

During late gestation, a portion of Pax3^+^/Pax7^+^ myogenic precursors migrate underneath fetal myofibers and become satellite cells at E16.5 (late gestation) in mice [75]. Satellite cells proliferate and fuse with existing myofibers to increase the nuclear density of each myofiber, which promote the synthesis of myofibrillar proteins and enlarge the diameters of myofibers [76]. After birth, satellite cells continue to enhance muscle hypertrophy and are responsible for myofiber regeneration in the case of an injury [76].

### Adipogenesis in different depots

Adipose tissues are generally divided into visceral, subcutaneous, intermuscular, and intramuscular adipose tissues, based on their locations [7]. In livestock, adipose of the visceral, subcutaneous, and intermuscular depots have low commercial values. Therefore, their accumulation significantly reduce feed efficiency. Intramuscular fat affects the palatability (flavor and juiciness) of meat, which in turn are associated with its commercial value. Therefore, it is essential to enhance intramuscular fat, while decreasing the relative abundance of fat in other depots, in order to enhance production efficiency and meat quality.

The development of adipose tissue is closely associated with angiogenesis, or the embryonic formation of circulatory vessels [77]. In ruminants and swine, “primitive fat organs” first appear with vascular structures in presumptive adipose depots with very limited adipocytes [77]. During fetal and early weaning stages, mature adipocytes are formed and rapidly increase their number by hyperplasia. This process is supported by angiogenesis [77]. Most adipocytes are formed before adolescence, and these numbers are maintained through consistent adipocyte turnover. Meanwhile, further expansion of adipose tissue is mainly due to adipocyte hypertrophy [78]. Therefore, the adiposity of animals can be manipulated by regulating the proliferation or differentiation of adipocytes depending on their developmental stages.

Interestingly, the development of adipose tissues at different depots shows a sequential order [7]. Subcutaneous fat develops first with its earliest precursors formed at the end of the first trimester of gestation [7, 44]. Subcutaneous adipocytes commit and differentiate during mid to late gestation, while their numbers remain quite stable postnatally [79, 80]. Visceral adipocytes develop later than subcutaneous adipocytes and mature during late gestation and after birth [79–81]. In beef cattle, intramuscular adipocytes expand primarily from the late gestation to around 250 days of age and undergo hypertrophy at the finishing stage. This active proliferating stage of intramuscular adipocytes is considered as the important “marbling window” which, provides an unique opportunity to enhance marbling in the meat without inducing overall adiposity of beef cattle [82]. In other words, by enhancing the maternal diet of pregnant beef cattle during this time, a farmer will “program” offspring to develop a higher ratio of desired intramuscular adipocytes compared to the aforementioned undesirable adipocyte depots.

### Fibrosis and adipogenesis from fibro/adipogenic progenitors

In addition to the structure of myofibrils, meat tenderness is also determined by the contents and cross-linking of connective tissue. While the toughness of meat caused by myofibrils can be partially resolved by postmortem changes, connective tissues are resistant to these changes and are responsible for background toughness [7, 83]. Controlling the development and differentiation of fibroblasts might help to solve toughness caused by connective tissue.

In adult skeletal muscle, fibroblasts and adipocytes derive from the same pool of multipotent progenitor cells, named as fibro/adipogenic progenitors (FAPs), which uniquely express platelet derived growth factor receptor α (PDGFRα) and stem cell antigen-1 (SCA-1). [84, 85]. Recent studies have partially unveiled the molecular mechanisms underlying the fate decision of FAPs in skeletal muscle under disease or dystrophic conditions [86]. For example, signaling of TGFβ and PDGF has been shown to induce the fibrotic differentiation of FAPs while signaling of WNT5A/GSK-3/β-catenin, Notch2, retinoic acid (RA) and IL4 restricts their adipogenic differentiation [87–91].

Embryonic FAP-like precursors have been identified in limb skeletal muscle at mid-gestation (E11.5-13.5) of mice with characteristic expression of odd skipped-related 1 (OSR1) [92]. These FAP-like cells support fetal myogenesis through the production of muscle-specific extracellular matrix components and the secretion of regulatory factors. A subset of OSR1^+^ precursors acquire SCA1 expression during late gestation and give rise to adult FAPs [92].

During adipose tissue development, adipogenesis and fibrogenesis work in unison to build the basic structure of adipose, as adipocytes find themselves embedded in a network of connective tissue. Lineage tracing studies show pools of collagen-expressing (Col1α1^+^) fibroblast precursors commit to either stromal fibroblast or preadipocytes in the embryos [93]. Overexpression of ZFP521 inhibits preadipocyte formation, while enhancing fibroblast population in early embryos [93]. In addition, PDGFRα^+^ precursors are also found in the mesenchyme of adult adipose tissue [94, 95]. Enhanced PDGFRα signaling in adipose tissue promotes their fibrotic fate decision [88, 96]. Moreover, a CD9^+^ subset of PDGFRα^+^ precursors secrete collagen and exhibit higher fibrotic potential than the other portions in adipose tissues [97]. High-fat diet feeding increases the ratio of the CD9^+^ PDGFRα^+^ subset and enhances fibrosis in adipose tissue [97].

As the density of FAPs determines the total number of cells for either adipogenic or fibrotic differentiation, there is a positive correlation between adipogenesis and fibrosis. Indeed, high-fat feeding of mice not only enhances the density of FAPs in skeletal muscle, but also promotes the accumulation of both lipids and fibrosis [89]. Maternal overnutrition in beef cattle enhances the contents of both fat and collagen in the skeletal muscle of their offspring [98]. In addition, 40% nutrient restriction in beef cows shows elevated gene expression of fibrogenic markers, suggesting enhanced accumulation of connective tissues [69]. Consistently, the genetically high-marbling Wagyu breed of cattle have more collagen accumulation in their skeletal muscle compared with those of other breeds [99]. On the other hand, the adipogenic or fibrotic fate decision of FAPs is a competitive process when the density of FAPs is defined. In this case, promoting adipogenesis of FAPs would decrease their fibrogenesis in skeletal muscle, thus improving marbling and meat tenderness. In summary, targeting the proliferation of FAPs and manipulating their fate decisions by nutritional methods would provide new opportunities to decrease connective tissue accumulation and enhance marbling in meat-producing animals.

### Maternal nutrition regulates fetal muscle development

Using the scaffold of primary myofibers, secondary myofibers are built between the 2 and 7 months of gestation in cattle [100]. After birth, the number of myofibers does not experience hyperplasia and skeletal muscle mainly grows in size and length by the contribution of satellite cells [68]. In addition to the active proliferation and differentiation of myoblasts that contribute to secondary myofiber formation, the fetal stage is also critical for the residency and proliferation of satellite cells [31]. Therefore, a reduction in the proliferation of fetal myoblasts and satellite cells decreases the number of fetal myofibers and the density of tissue-resident stem cells which negatively affect the development and growth of skeletal muscle after birth [101–103]. Consistently, 50% nutrient deficiency during the mid to late gestation (28–78 dG) of ewes impaired the formation of secondary myofibers [104], which was correlated with the reduced mass of myofibers, and their numbers, in offspring lambs [101]. Maternal 60% caloric restriction in beef cattle during 30–140 dG in beef cattle reduced the size of fetal myofibers at 140 dG [69]. In addition, both restricted (60% of National Research Council, NRC, 2007 [105]) or overfed (140% NRC) ewes during 30–90 dG reduced the ratio of secondary to primary myofibers, and the percentage of PAX7^+^ myogenic progenitors in the fetal muscle on 90 dG [106]. Moreover, maternal over-nutrition was found to enhance NF-κB signaling pathways in the fetal skeletal muscle of ewes, which downregulated WNT/β-catenin and reduced myogenic gene expression [107]. Therefore, both nutrient deficiency and overfeeding during fetal myogenesis reduce the number of myofibers, the mass of skeletal muscle and the density of satellite cells, which exhibit long-term effects on muscle growth in offspring.

### Maternal nutritional regulates satellite cell density and lean growth

After the formation of secondary myofibers, satellite cells fuse with existing myofibers and contribute to the growth of myofibers in size and the density of nuclei [76]. Therefore, the size, but not the number, of myofibers is affected by maternal nutrient fluctuations during this period. The density of satellite cells formed at this stage also determines the potential of muscle growth after birth. In sheep or cattle with twin pregnancies, the two enlarging fetuses compete for the limited amount of nutrients present during late gestation. This indirectly results in relative nutrient deficiency compared to singleton pregnancy. Impaired growth performance and decreased fetal skeletal muscle, but not the number of myofibers, were observed in the offspring of twin pregnancies [108, 109]. In addition, protein supplementation during 140 to 254 dG in beef cattle increases the cross-sectional areas of myofibers [69]. Calf birth weight and growth performance of offspring steers were also improved by nutrient supplementation during the late gestation in a winter grazing system [110]. In contrast, nutrient restriction during the last two thirds of pregnancy reduced the weight of neonatal calves [111]. Interestingly, vitamin A administration at this neonatal stage increased the density of PAX7^+^ satellite cells, myogenesis, and the ratio of oxidative myofibers in calves at 2 months of age [13]. Therefore, enhancing the density of satellite cells during their active proliferating stages increases the potential of muscle growth during the adult stages of offspring.

### Maternal nutrition affects myofiber composition

In rodents and swine, muscle fibers are mainly composed of slow-twitch type I fibers, fast-twitch type IIa myofibers, and fast-twitch type IIb myofibers according to their contractile properties [74]. In ruminants, type IIx replaces type IIb as the dominant fast twitch type. While type I myofibers primarily utilize oxidative phosphorylation for their metabolism, type IIb myofibers mainly use glycolysis. Metabolism in type Ila is a mixture of both oxidative phosphorylation and glycolysis. Though the relative ratio of myofibers with different metabolic properties is determined by genetic factors, it is also susceptible to physiological and nutritional alterations [112]. Fast-growing animals are characterized with increased ratios of glycolytic myofibers [113, 114]. Increased dietary energy levels are also positively related to the switch of myofiber metabolism from oxidative phosphorylation to glycolysis in beef cattle [115, 116]. The ultimate postmortem pH is heavily affected by the high glycogen contents in the glycolytic myofibers, which reduces water holding capacity and juiciness of meat.

The metabolic phenotype of myofibers in offspring is correlated with changes of maternal nutrition. 50% nutrient deficiency during 30 to 70 dG in ewes reduced the number of fast-twitch myofibers while increasing the number of slow-twitch myofibers in the newborn lambs [117]. However, at 10 months of age, offspring born to nutrient-restricted dams had a higher ratio of fast-twitch myofibers [101]. In contrast, a maternal high-fat/low-fiber diet of pigs starting from 60 dG decreased mitochondrial function and elevated protein expression of fast-twitch MHC IIb and IIx in skeletal muscle of neonatal offspring [118]. Therefore, the level of maternal nutrition has significant effects on muscle composition and meat quality of offspring.

### Maternal nutrition during fetal and neonatal stages regulates adipogenesis and fibrosis

Adipocytes and fibroblasts in the skeletal muscle derive from the adipogenic or fibrogenic differentiation of FAPs. In beef cattle, the proliferation of FAPs occurs from the fetal and neonatal stages followed by the weaning stage to about 250 days of age [7]. After that, FAPs mainly remain quiescent under a normal physiological condition and their density tends to decline with aging. Therefore, enough intramuscular adipocytes form during their active proliferating stages ensure their effective differentiation at later stages. Consistently, beef cattle are fed with high-grain diet during the finishing stage to induce the hypertrophy of intramuscular adipocytes [119]. However, the density of FAPs differs among animals of different breeds, which affects the effectiveness of adipogenic differentiation of FAPs during the fattening stage.

Through nutritional supplementation during their active proliferating stages, the density of FAPs can be enhanced [11]. However, the proliferation of visceral and subcutaneous adipocytes is also active during the fetal and neonatal stages. Fluctuations in maternal nutrition during this period affect the overall adiposity of animals. Indeed, 20% reduction of maternal energy during the fetal stage increased the 12^th^ rib fat thickness as well as the intramuscular fat of cows [120]. Maternal nutrient restriction during 28–80 dG in ewes led to increased mass of fetal perirenal adipose tissue and higher *Pparγ* gene expression [121]. On the other hand, increased dietary protein levels in the pasture during the fetal stage of beef cattle decreased the share force of the meat while elevating the 12^th^ rib fat thickness (reflecting overall fatness) and the density of subcutaneous adipocytes in offspring steers [122]. Consistently, 150% over-nutrition from 60 d before conception of ewes to birth enhanced not only the content of intramuscular fat but also the content and cross-linking of collagen in the skeletal muscle of adult offspring [123, 124]. In addition, the smallest piglet in a litter which had experienced nutrient deficiency during gestation showed higher proportion of adiposity and collagen I content compared to the largest piglet [125]. To avoid increasing the adipogenesis of subcutaneous and visceral fat, nutrient supplementation can be provided during the early weaning to about 250 days of age in beef cattle when only intramuscular adipocytes are actively forming. Consistently, the contents of intramuscular fat are increased in early weaned beef cattle fed with a high-grain diet [126]. Grass-fed beef generally contain low marbling because of the absence of a high-grain diet during the finishing stage [127].

The Japanese Black cattle are well known for their high marbling, with more than 30% intramuscular fat [128]. Decreased vitamin A feed after 14 or 15 months of age enhances marbling compared to those fed a high vitamin A diet [129, 130]. This is linked to gradually reduced IGF-1 concentration in the serum with low vitamin A intake [129]. However, low or deprived vitamin A diet induces multiple negative outcomes such as the occurrence of blindness and muscular edema, severe hepatic disease, and swelling. Therefore, currently, farmers are limiting the vitamin A concentration only during the middle fattening period to avoid severe side effects [128]. Thus, vitamin A restriction is used to increase marbling in finishing beef cattle [131]. On the other hand, vitamin A administration during neonatal stages increase intramuscular fat content by 45%, despite not increasing overall fatness [12, 132]. Consistently, one single shot of vitamin A at birth also enhanced marbling of beef cattle at harvest [133]. This increase in marbling is mainly due to enhanced hyperplasia of PDGFRα^+^ FAPs through RA-enhanced VEGFRα-mediated angiogenesis [12, 132, 133]. We recently investigated the role of RA-signaling in the cellularity of FAPs using a mouse model with blockage of RA-signaling specific in FAPs [89]. Our results showed that RA signaling is indispensable for the maintenance of FAP stemness and elevated-RA signaling promotes the hyperplasia of FAPs. RA binds to the retinoic acid X receptor (RXR) and partners with PPARγ, which is required for the early stage of adipogenesis, while stimulates lipid oxidation through activation of PPARα and β/δ in mature adipocytes, generating stage-specific effects in increasing adipocyte hyperplasia but suppressing hypertrophy [134].

### Developmental programming in poultry

Besides mammals, previous studies have also demonstrated the dramatic effects of embryonic development on the health and productivity of birds [135, 136]. Avian embryos are accessible, and the embryonic environment can be readily manipulated by in ovo administration of prospective materials [137]. The amnion has been considered as the ideal site for delivery of external materials although delivery to the other sites including the allantoic membrane, the air cell, the yolk sac, and the embryo body were also reported [136, 137]. Over the last few decades, multiple vaccines have been delivered to the late-stage embryos to protect birds against diseases such as Marek’s disease, infectious bursal disease virus, fowl pox, and others [136–139]. In ovo injection of prebiotics/probiotics and supplemental nutrients including amino acids, vitamins, and nucleotides have also been used in birds to improve intestinal health, enhance immunity, and prevent various infections [140–144]. Bone strength could be potentially enhanced by the administration of trace minerals and vitamin D3 [145]. In addition, multiple studies also reported that in ovo feeding of egg white protein, amino acids, carbohydrates, creatine pyruvate, *L*-carnitine and other nutrients increased the body weight and pectoralis muscle mass in turkey or broilers [144, 146–150]. Administration of prebiotic and symbiotic not only enhanced the immunity but also increased the weight of the pectoral muscle while decreasing the collagen content of broiler chickens [144]. Therefore, developmental programming is also applicable to poultry and related studies will benefit the poultry industry.

### Maternal nutrition affects meat quality

As stated above, maternal nutrition affects the composition of myofibers as well as the formation of both intramuscular fat and connective tissues within the skeletal muscle, which ultimately leads to changes in meat quality. Indeed, improved pasture during the mid to late gestation of cows decreased Warner–Bratzler shear force in offspring steers finished to slaughter weight [21]. Similarly, protein supplementation during the mid-gestation decreased the tenderness of steaks produced by offspring with increased fatty acid content [151]. Protein supplementation during the late gestation also increased marbling scores and the grading of beef meat [152]. In contrast, some other studies reported that energy restriction or reduced protein supplementation during the mid or late gestation also has the potential to enhance marbling score and the accumulation of intramuscular lipids [120, 151, 153]. Therefore, while it is clear that maternal nutrition has significant effects on muscle composition and meat quality, the exact effects of different nutritional management and the timing of effective intervention await further investigation.

## Conclusion

Growing evidence suggests that nutritional manipulations not only during the fetal stages, but also during early embryonic stages, have profound effects on animal growth and development in offspring. Compared to vital organs like the brain, the development of skeletal muscle, adipose tissue, and connective tissue is more sensitive to maternal nutritional fluctuations. Nutritional variations induce changes in the formation of early myofibers and adipocytes/fibroblasts, and the number of tissue resident progenitors, which generate long-term alteration in body composition, growth characteristics, and meat quality of offspring. More studies are needed to understand the maternal impacts on prenatal animal development, especially during the early embryonic stage, which sets the trajectories for later fetal and postnatal development. Understanding these underlying regulatory mechanisms will help us to develop better strategies to enhance livestock production efficiency and meat quality through stage-specific nutrient supplementations.

## Data Availability

Not applicable.

## Abbreviations

d: Day/days
dG: Days of gestation
DM: Dermomyotome
E: Embryonic day
FAPs: Fibro/adipogenic progenitors
LPM: Lateral plate mesoderm
MHC: Myosin heavy chain
NOTO: Notochord
NRC: National Research Council
NTB: Neural tubes
RA: Retinoic acid
SCL: Sclerotome
